# Empirical Validation of Pooled Whole Genome Population Re-Sequencing in *Drosophila melanogaster*

**DOI:** 10.1371/journal.pone.0041901

**Published:** 2012-07-26

**Authors:** Yuan Zhu, Alan O. Bergland, Josefa González, Dmitri A. Petrov

**Affiliations:** 1 Department of Genetics, Stanford University, Stanford, California, United States of America; 2 Department of Biology, Stanford University, Stanford, California, United States of America; 3 Institute of Evolutionary Biology (Spanish National Research Council-Pompeu Fabra University), Passeig Maritim de la Barceloneta, Barcelona, Spain; Seoul National University College of Medicine, Korea

## Abstract

The sequencing of pooled non-barcoded individuals is an inexpensive and efficient means of assessing genome-wide population allele frequencies, yet its accuracy has not been thoroughly tested. We assessed the accuracy of this approach on whole, complex eukaryotic genomes by resequencing pools of largely isogenic, individually sequenced *Drosophila melanogaster* strains. We called SNPs in the pooled data and estimated false positive and false negative rates using the SNPs called in individual strain as a reference. We also estimated allele frequency of the SNPs using “pooled” data and compared them with “true” frequencies taken from the estimates in the individual strains. We demonstrate that pooled sequencing provides a faithful estimate of population allele frequency with the error well approximated by binomial sampling, and is a reliable means of novel SNP discovery with low false positive rates. However, a sufficient number of strains should be used in the pooling because variation in the amount of DNA derived from individual strains is a substantial source of noise when the number of pooled strains is low. Our results and analysis confirm that pooled sequencing is a very powerful and cost-effective technique for assessing of patterns of sequence variation in populations on genome-wide scales, and is applicable to any dataset where sequencing individuals or individual cells is impossible, difficult, time consuming, or expensive.

## Introduction

Efficient assessment of presence and frequencies of single-nucleotide polymorphisms (SNP) in populations is vital to answering key problems in genetics and population biology. For instance, inference of demographic history, identification of causative loci affecting a trait of interest, discovery of cancer-causing mutations in mixed pools of cells, or the search for evidence of natural selection in the genome all require knowledge of the frequency spectra in groups of individuals or cells. However, individually sequencing dozens of individuals from each population is often more costly and labor intensive. Multiplexing techniques allow a more efficient use of sequencing resources but still require a large number of individual DNA extractions, manipulations of reagents, barcoding oligos, PCR reactions, and sequencing library constructions. The same applies to overlapping pools of non-indexed samples [1]. In contrast, pooling individuals prior to DNA extraction and sequencing the pooled DNA without barcodes can generate an inexpensive and efficient assessment of allele frequencies genome-wide.

The empirical accuracy of pooled re-sequencing has been assessed in small genomes or small genomic regions re-sequenced to 20–8,800× coverage [2]–[9]. In prokaryotes, pooling has been applied to clonal salmonella populations [4]. In eukaryotes, pooled Reduced-Representation Libraries (RRL) that cover a smaller portion of the whole genome have been re-sequenced for cattle [3] and domesticated pig [8]. Pooling has also been tested with individual human genes [6], [9] and applied to disease studies where the genomic regions of interest in affected patients were re-sequenced in pooled samples to detect disease related variants [2], [5], [7].

While genome-wide pooled re-sequencing has been applied to *Drosophila melanogaster* [10]–[12], *Arabidopsis lyrata* [13], and *Anopheles gambiae* [14], the accuracy of allele frequency estimation was not assessed in these studies. There are two reasons why pooled sequencing of whole eukaryotic genomes might result in less accurate frequency estimates than smaller genomes or small genomic regions. First, lower coverage per chromosome and increased genetic complexity may increase mismapping around structural variations. Second, unequal contributions of DNA from each individual may systematically bias allele frequency estimates [15].

We generated a series of pooled re-sequencing libraries of *D. melanogaster* and show here that pooled re-sequencing provides a highly accurate assessment of allele frequencies genome-wide. When a sufficient number of chromosomes are pooled, the resulting allele frequency estimates are not biased by unequal DNA contributions and can be well approximated by a simple binomial distribution that depends only on read depth and allele frequency. Our results imply that pooled whole genome population sequencing should be easily applicable to any organism whose genome can be sequenced to ∼50–100× coverage. Currently, this limits the technique to organisms with small to moderate genome sizes. However, the continued increases in sequencing throughput will make the pooled re-sequencing approach relevant for organisms with larger genomes such as humans and many crop plants.

## Methods

### Drosophila Strains

**Figure 1:**
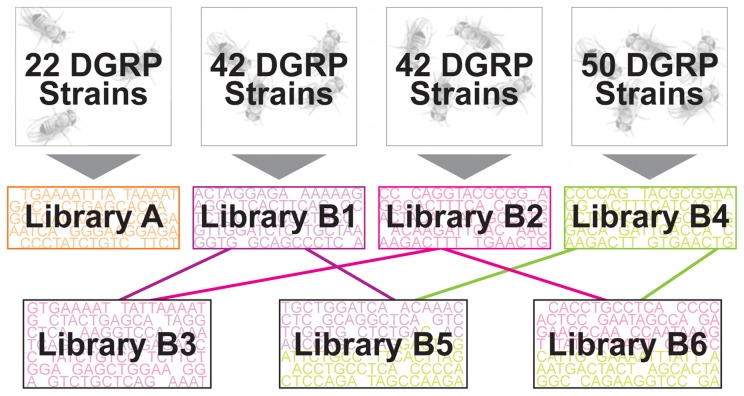
Schematic representation of the pooled libraries used in this study. Libraries A, B1, B2 and B4 were constructed pooling flies from different number of DGRP strains. Libraries B3, B5 and B6 are the result of merging reads from libraries B1, B2 and B4.

All isogenic fly strains came from the Drosophila Genetic Reference Panel (DGRP) [16] and were maintained in our laboratory. We picked 112 out of the total 192 strains included in the DGRP as follows: we picked 22 DGRP strains that were also independently sequenced by the Drosophila Population Genomics Project DPGP at UC Davis (http://www.dpgp.org/) to make library A (Figure 1; Supporting Information S1). Another 42 DGRP strains were randomly picked to make libraries B1 and B2, and a final 50 DGRP strains (mutually exclusive from the 42) went into library B4 (Figure 1; Supporting Information S1).

### True Frequency Estimation

True DGRP source population allele frequencies were obtained from the 162 sequenced DGRP strains. Only sites with no residual within strain heterozygosity within the 162 DGRP strains were used to assess the accuracy of pooled resequencing.

### Library Construction

Library A (SRR353364.1) was constructed from a pool of 220 flies (10 females per strain) with DNA extracted using the Qiagen Gentra Puregen tissue kit. Library A was sequenced on a single lane of Illumina GAIIx to a total depth of 10× with 100 bp paired-end reads. Libraries B1, B2 and B4 (SRR353365.1) were constructed from pools of male flies (1 male per strain) with DNA extracted via isopropanol precipitation [17]. Libraries B1, B2 and B4 were sequenced on a single lane of Illumina HighSeq 2000 to a total depth of 60× (20× each). Reads were 93 bp paired-end after accounting for barcode length.

Reads from libraries B1 and B2 were merged to “construct” B3 library (Figure 1). Library B3 has therefore the same number of strains as B1 and B2 (i.e. 42 strains) but twice as many flies pooled and double the read depth coverage of B1/B2. Finally reads from libraries B1 and B2 were merged independently with reads from library B4 to construct two 92 strains pooled libraries: B5 and B6 respectively (Figure 1).

### Mapping/SNP Calling

All reads were mapped to the *D. melanogaster* reference genome release 5.33 using default parameters. No read trimming was done. Read pairs where one read was unmapped were discarded. Final analysis was restricted to positions covered to a minimum of 10× in all libraries. Initial alignments for both DPGP and pooled libraries were carried out with BWA (version 0.5.9) post-processed with samtools (version 0.1.18) [18]. Downstream base quality score recalibration, indel realignment, and SNP discovery [19] were carried out in GATK (version 1.2 for DPGP and 1.4 for pooled libraries) [20]. Library A was also separately mapped with MAQ (version 0.7.1) [21]. Allele frequency estimates were calculated as the percentage of reads carrying the non-reference allele at a DGRP identified polymorphic site. Novel SNP discovery in library B6 was carried out using GATK Unified Genotyper command using default parameters.

### Accuracy estimates

Two values were computed as accuracy measures of allele frequency estimates from pooled libraries. Concordance correlation was computed using the epiR package (http://epicentre.massey.ac.nz) version 0.9–32. Relative error were computed according to the formula Error = ((freq_pool_−freq_DGRP_)/freq_DGRP_)∧2.

### Binomial Simulations

Simulations were carried out in R (version 2.13.0) [22]. For each library, 1,000 SNPs were randomly selected (all SNPs were binned by frequency into 20 bins in 5% increments, and 50 SNPs were selected randomly from each bin per run) to obtain 1 observed concordance correlation and 1 relative error value. Binomial simulation based on observed read depth distribution from real sequencing data was then run on the set of 1,000 SNPs to obtain binomial concordance correlation and relative error values. This was repeated 100 times to obtain observed and simulated concordance and error rates.

## Results

We designed and constructed a series of pooled resequencing libraries (SRA046699.1) from varying numbers of isogenic *D. melanogaster* strains from the DGRP panel (Table 1 and Figure 1) and compared the accuracy of allele frequency estimates from our pooled libraries to simulated expectations. We investigated the effects of mapping strategies, read depth, unequal DNA contribution, and reproducibility of the technique with regards to the accuracy of population allele frequency estimation from pooled sequencing. Finally, we estimated the efficiency of novel SNP discovery from pooled libraries.

**Table 1:** Pooled Libraries.

Platform	Sex	Read Length	Library	Strains Pooled	Flies Pooled	Read Depth
GAIIx	F	100 bp PE	A	22	220	10×
Hi-Seq 2000	M	93 bp PE	B1	42	42	20×
Hi-Seq 2000	M	93 bp PE	B2	42	42	20×
Hi-Seq 2000	M	93 bp PE	B3	42	84	40×
Hi-Seq 2000	M	93 bp PE	B4	50	50	20×
Hi-Seq 2000	M	93 bp PE	B5	92	92	40×
Hi-Seq 2000	M	93 bp PE	B6	92	92	40×

### Allele frequency estimation: sources of error

#### Mapping tools

We first tested if the choice of mapping tools affects the accuracy of allele frequency estimation. We began by creating pooled library A from 22 DGRP strains. We remapped library A in 3 ways - MAQ, BWA/Samtools, and BWA/Samtools+GATK. We looked at DGRP SNP positions that were also covered to > = 10× read depth in library A and compared DGRP allele frequency estimates (from all 162 strains) to those from library A. To account for bias caused by a proportionally larger number of low frequency SNPs, we randomly selected 1000 SNPs in each of 100 runs, with evenly distributed SNP allele frequencies in each 1000 SNP set, for analysis and simulation of expected concordance with DGRP allele frequencies given pool coverage. The results obtained were roughly comparable across mapping tools (concordance correlations: MAQ = 0.701–0.783, BWA = 0.823–0.76, BWA+GATK = 0.822–0.867) and should uniformly affect all pooled libraries as well as individual sequencing (Table 2). BWA+GATK were used for all subsequent analyses.

**Table 2:** Library allele frequency estimate comparison to 162 DGRP strains.

Library	Mapping Tool	Compared To	Observed Concordance	Expected Concordance	Observed Relative Error	Expected Relative Error
A	MAQ	161 DGRP	0.701–0.783	0.934–0.952	0.3748–2.031	0.2022–1.317
A	BWA	161 DGRP	0.823–0.876	0.931–0.952	0.308–1.677	0.165–1.479
A	BWA,GATK	161 DGRP	0.822–0.867	0.931–0.952	0.325–2.471	0.184–1.174
B1	BWA,GATK	161 DGRP	0.906–0.934	0.933–0.952	0.278–6.713	0.182–1.025
B2	BWA,GATK	161 DGRP	0.911–0.936	0.930–0.950	0.215–4.932	0.175–1.258
B3	BWA,GATK	161 DGRP	0.921–0.939	0.935–0.954	0.211–7.179	0.202–1.293
B4	BWA,GATK	161 DGRP	0.918–0.931	0.932–0.944	0.361–3.806	0.193–1.152
B5	BWA,GATK	161 DGRP	0.932–0.954	0.944–0.962	0.198–4.074	0.159–1.263
B6	BWA,GATK	161 DGRP	0.934–0.955	0.945–0.963	0.152–4.146	0.177–1.971

#### Read depth

The observed concordance correlations of library A allele frequency estimates as compared to ‘true’ DGRP source population allele frequencies, regardless of mapping tool, were >10% lower than binomially simulated values (Table 2). We needed higher coverage libraries to explore the possibility that increasing coverage further will place observed correlations within expectations.

We followed up with a series of B libraries (Fig. 1). These libraries were made from different subsets of DGRP strains that mimic independent samples from the same source population as DPGP strains, but with true genotypes unknown, mimicking real experiments where samples would be collected from populations with unknown true allele frequencies. As in library A, we used all 162 DGRP genotypes for estimates of true population allele frequencies. Libraries B1 and B2 (Table 1 and Figure 1) were independent collections of flies pooled from the same 42 DGRP strains, individually processed during DNA extraction and library making and sequenced to 20× coverage. We placed minimum (>10×) read depth filters as with library A, and observed concordance correlation values of (0.906–0.934) and (0.911–0.936) for B1 and B2 respectively. These genome-wide correlations were just below the binomial correlations of (0.933–0.952) and (0.930–0.950). This was a marked improvement from library A where we simulated binomial values between (0.931–0.952) but observed (0.822–0.867) with 10× read depth requirements (Table 2). However, these libraries, being pools of twice as many strains as library A, were not directly comparable to tease out the effects of coverage. We thus combined libraries B1 and B2 to ‘construct’ library B3 so as to compare libraries B3 to B1 and B2 as libraries with the same number of pooled strains, but with library B3 containing twice as many reads as libraries B1/B2 (Table 1). The correlation between actual and observed allele frequencies for library B3 (0.921–0.939) was <2% lower than binomial (0.935–0.954). The accuracy of allele frequency estimation in library B1 and B2 were also ∼2% lower than binomial. Thus, the two-fold change in coverage between B3 and B1 and B2 did not substantially change the accuracy of allele frequency estimates. Library B4 that was pooled independently from 50 male flies (comparable to libraries B1 and B2) each representing a different DGRP strain not used in the making of B1 and B2 (Table 1 and Figure 1) and sequenced to a similar read depth (20×), was similarly ∼2% lower than binomial (Table 2).

#### Unequal DNA contribution

**Figure 2:**
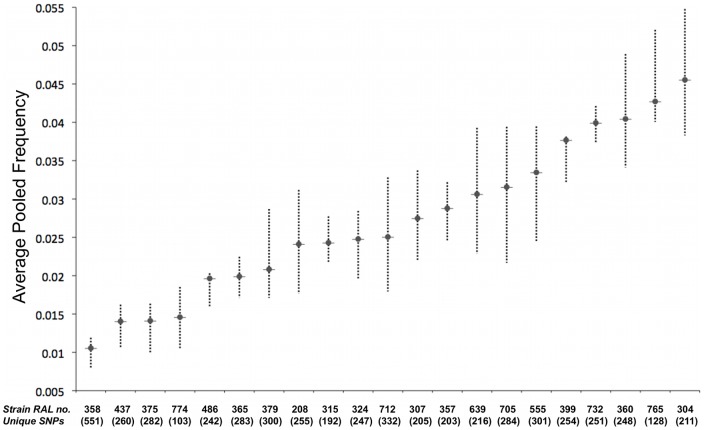
DNA contribution of strains in Library A. DNA contributions of each strain as observed from representation of unique SNPs in pool. Each vertical line represents pooled frequency estimates of singleton SNPs unique to a single strain. Edges of lines represent values at 2 standard deviations from mean (thick horizontal line). Strain RAL-301 was dropped from analysis due to low unique SNP count. Y-axis: DNA contribution in the form of pool frequency estimate from library A. X-axis: Strains sorted in order of mean DNA contribution.

We explored the possibility that unequal DNA contribution from pooled strains leading to over-representation of SNPs present in strains with larger flies or flies generating more DNA for some other reason, was the larger source of noise. Library A was suitable for this analysis because 10 flies were used from each strain, and should amplify any effects from variance in DNA material. The 22 strains used in library A were fully sequenced by DPGP. Strain RAL-301 had significantly fewer unique SNPs compared to all other strains, partially due to its low coverage in DPGP data, and was dropped from the analysis. Using polymorphic position identified by DGRP, we identified approximately 250 ‘private SNPs’ unique to each of the remaining 21 strains in library A (Figure 2). SNPs were identified as unique to a strain if a DGRP SNP position was covered to >4× in all 21 strains, was found fixed in just 1 strain while being absent in all others, and covered to >10× in library A. 50 SNPs were randomly picked from each strain and their average frequency as estimated through library A calculated. This was repeated 10 times to obtain mean pool frequency and 2 standard deviation values. The average frequencies of these singleton SNPs within the pooled library A were treated as good approximations for the relative DNA contributions of each strain. We found that there was a gradation in DNA representation from each strain (Fig. 2), suggesting that unequal DNA contribution was a significant issue in our pool. However, the distribution of the relative DNA contributions from each strain is gradual without a clear divide between over-represented and under-represented strains. This implies that the variation is likely a by-product of sampling and not due to PCR jackpotting and therefore should be easily remedied by pooling more strains together.

**Figure 3:**
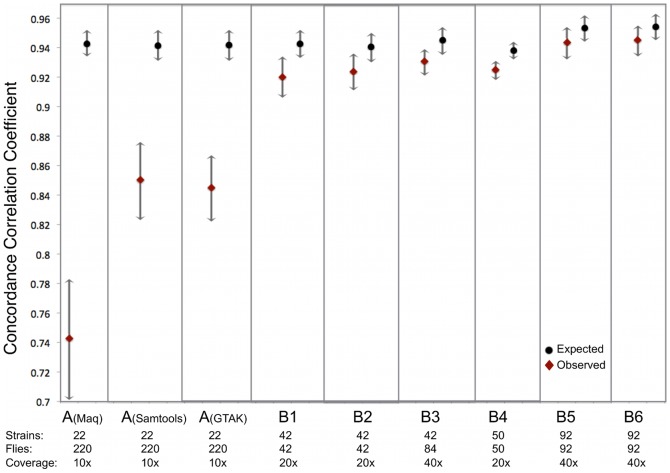
Expected and observed pooled frequency estimates. Correlation coefficients between observed or simulated pooled allele frequency estimates and actual estimates as a function of the number of strains pooled. See Materials and Methods for a description of the libraries. Y-axis: Expected (triangles) and observed (circles) correlation coefficients of the 7 libraries compared to a perfectly binomial library, color-coded by library. X-axis: Libraries ordered by number of strains pooled.

To test this prediction, we analyzed the B libraries that vary in the number of strains used. Previously, the use of singleton SNPs to estimate DNA contributions was possible in library A because all 22 strains pooled were individually sequenced by DPGP. However, we were not able to perform the same analysis for the B libraries because whole genome sequences for some of the strains were not available. Thus, in order to test the effects of the number of input strains on allele frequency estimates, we contrasted the expected and observed accuracy of allele frequency estimates from libraries B1–B6 (Table 2). Libraries B1 and B2 contain 42 strains sequenced to ∼20× coverage, libraries B3 and B4 contain 42 and 50 strains respectively sequenced to ∼40× coverage and libraries B5 and B6 contain 92 strains sequenced to ∼40× coverage. The accuracy of allele frequency estimates of libraries B1–4 are ∼1–3% lower than expected. However, the accuracy of allele frequency estimates of libraries B5–6, (0.932–0.954) and (0.934–0.955) respectively, largely overlap with binomial values (0.944–0.962) and (0.945–0.963) (Figure 3). Thus, the number of strains used to make sequencing pools appears to be a major source of error in allele frequency estimation.

### Allele frequency estimation: reproducibility

Libraries B1 and B2 were designed specifically to estimate the effect of technical and biological error on pooled frequency estimates. As biological replicates, they allowed us to estimate the compounded error introduced into final allele frequency estimates from unavoidable technical and biological error including variability in fly rearing, pooling, DNA extraction, PCR reactions, and sequencing. We expected that if there were no external sources of error from DNA extraction and library making protocol, B1 and B2 should behave as two independent binomial samples from the same source population. Simulations place expected correlation of B1 to B2 at (0.903–0.909), while observed correlation coefficient was 0.898. The correlation between B1 and B2 allele frequency estimates is closer to binomial expectations than the correlations between B1/B2 with the DGRP and their respective binomial expectations. As the only differences between libraries B1 and B2 were the flies used (different flies from the same strains), this suggests that compounded error from experimental components such as library making, pooling, and mapping is small relative to error from unequal DNA contribution from different flies.

### SNP discovery

**Figure 4:**
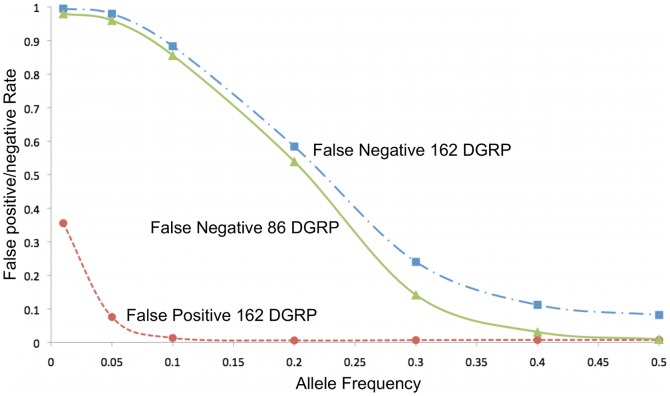
SNP Discovery Error Rates. False positive and false negative rates of novel SNP discovery from pooled library B6. Y-axis: Error rate. Z-axis: Folded allele frequency from DGRP genotypes.

In addition to allele frequency estimation, novel SNP discovery from pooled libraries is an important application for population sequencing. In real life experiments where it is usually impossible to sample the full set of variants in a population and false negative rates are inherently high, it is more important that the SNPs that are called are real. To assess false positive rates of SNP calling, we compared our SNP calls from library B6 to the 162 sequenced genotypes from DGRP database, sorted by allele frequency (Fig. 4). As expected, false positive rate is higher for rare alleles but quickly falls to 7% for alleles present at >5% and around 1% for those present at >10% in the population. False negative rates, also included, might be exaggerated in this dataset as fewer SNPs are actually present in the pool of 92 strains than there are in the source population of 162 strains. We thus also calculated false negative rates using the 86 overlapping strains between DGRP and our pool, and observed improvements in high frequency alleles.

## Discussion

We resequenced a series of pooled libraries made from largely isogenic, individually sequenced *D. melanogaster* strains. Allele frequency estimates derived from the pooled libraries were compared to corresponding estimates derived from individually sequenced strains (exact) or the source population (estimate). While there was significant variance in relative DNA contribution from the smallest pool of 22 strains, and association between pooled allele frequency estimates and true allele frequencies was lower than expected under a binomial model, this discrepancy disappeared as an increasing number of strains were pooled. Given sufficient number of strains pooled, we found that pooled sequencing provides an accurate estimate of population allele frequency with the error well approximated by binomial sampling. We focused on allele frequencies of known SNPs because our goal was to verify that pooled libraries are true representations of the sampled population. However, pooled libraries are also effective means of calling new SNPs without the redundancy of sequencing multiple individuals [15]. We found that SNP discovery had low false positive rates and was effective for common variants in the population.

Pooled libraries can also be used to estimate frequency of transposable element insertions [23, Fiston–Lavier et al personal communications], used in the estimation of Θπ, ΘS, and Fst [11], [15], and combined with likelihood methods for more powerful estimation of population genetics parameters such as nucleotide diversity and allele frequency [24]–[27], which are important for species where nucleotide diversity is low (such as humans) and more affected by high-throughput sequencing errors [28]–[31]. Pooled sequencing data can also be used in detecting selective sweeps [32], and could yield limited information on linkage as haplotype information is retained on the order of read lengths and the distance covered by each paired end reads. As statistical methods are constantly refined to deal with the complexities of pooled data [6], [33]–[38], our power to analyze such data will increase.

Pooled libraries are a means of efficiently sampling genetic variation in any group of polymorphic chromosomes. Possible applications of pooled re-sequencing include complex tissue samples from diseased cells, populations under artificial selection, samples of wild caught individuals, and endosymbionts or mitochondrial genomes [39]. In essence any dataset where sequencing individuals is impossible, difficult, time consuming, or just expensive are prime candidates for the use of pooled sequencing. More importantly, given our result that pooling approximates binomial sampling given a sufficient number of pooled chromosomes, it is easy to estimate the required coverage for the desired power of each analysis.

This method of estimating required samples or coverage for an experiment is theoretically applicable to any species, tissue type, or chromosome with known genomic/molecule size and a reasonable reference sequence. Even though most linkage information is lost, population allele frequencies can be sampled over a larger number of individuals at a fraction of the cost of individual sequencing. Given current sequencing power (which is likely to increase further in the near future) pooling would be easily applicable to any organism with euchromatic genome sizes less than ∼500 Mb, including many model and non-model organisms. The continued increases in sequencing throughput will make pooled sequencing relevant for organisms with larger genomes such as humans and many crop plants. We believe that pooling is a very powerful and cost-effective technique for detecting unusual genomic patterns in populations on genome-wide scales.

## Supporting Information

Supporting Information S1 **Strains used in libraries pooled.**
(DOC)

## References

[1] Erlich Y, Chang Kenneth, Gordon A, Ronen R, Navon O, et al (2009) DNA Sudoku – harnessing high-throughput sequencing for multiplexed specimen analysis. Genome Res 19: 1243–1253. 19447965 10.1101/gr.092957.109 PMC2704425

[2] Shaw SH, Carrasquillo MM, Kashuk C, Puffenberger EG, Chakravarti A (1998) Allele frequency distributions in pooled DNA samples: applications to mapping complex disease genes. Genome Res 8 2 111–23. 9477339 10.1101/gr.8.2.111

[3] Van Tassel CP, Smith TP, Matukumalli LK, Taylor JF, Schnabel RD, et al (2008) SNP discovery and allele frequency estimation by deep sequencing of reduced representation libraries. Nature Methods 5 3 247–52. 18297082 10.1038/nmeth.1185

[4] Holt KE, Teo YY, Li H, Nair S, Dougan G, et al (2009) Detecting SNPs and estimating allele frequencies in clonal bacterial populations by sequencing pooled DNA. Bioinformatics 25 16 2074–2075. 19497932 10.1093/bioinformatics/btp344 PMC2722999

[5] Hajirasouliha I, Hormozdiari F, Sahinalp SC, Birol I (2008) Optimal pooling for genome re-sequencing with ultra-high-throughput short-read technologies. ISMB 24: i32–i40. 10.1093/bioinformatics/btn173 PMC2718651 18586730

[6] Out AA, van Minderhout IJ, Geoman JJ, Ariyurek Y, Ossowski S, et al (2009) Deep sequencing to reveal new variants in pooled DNA samples. Hum Mutat 30 12 1703–12. 19842214 10.1002/humu.21122

[7] Bansal V, Harismendy O, Tewhey R, Murray SS, Schork NJ, et al (2010) Accurate detection and genotyping of SNPs utilizing population sequencing data. Genome Res 20 4 537–545. 20150320 10.1101/gr.100040.109 PMC2847757

[8] Amaral AJ, Ferretti L, Megens H-J, Crooijmans RPMA, Nie H, et al (2011) Genome-wide footprints of pig domestication and selection revealed through massive parallel sequencing of pooled DNA. PLoS ONE 6 4 e14782. 21483733 10.1371/journal.pone.0014782 PMC3070695

[9] Margraf RL, Durtschi JD, Dames S, Pattison DC, Stephens JE, et al (2011) Variant identification in multi-sample pools by Illumina genome analyzer sequencing. Journal of Biomolecular Techniques 22: 74–84. 21738440 PMC3121147

[10] Burke MK, Dunham JP, Shahrestani P, Thornton KR, Rose MR, et al (2010) Genome-wide analysis of a long-term evolution experiment with Drosophila. Nature 467: 587–590. 20844486 10.1038/nature09352

[11] Kolaczkowski B, Kern AD, Holloway AK, Begun DJ (2011) Genomic differentiation between temperate and tropical Australian populations of Drosophila melanogaster. Genetics 187: 245–260. 21059887 10.1534/genetics.110.123059 PMC3018305

[12] Turner TL, Stewart AD, Fields AT, Rice WR, Tarone AM (2011) Population-Based Resequencing of Experimentally Evolved Populations Reveals the Genetic Basis of Body Size Variation in *Drosophila melanogaster* . PLoS Genet 7 3 e1001336 doi:10.1371/journal.pgen.1001336. 21437274 10.1371/journal.pgen.1001336 PMC3060078

[13] Turner TL, Bourne EC, Von Wettberg EJ, Hu TT, Nuzhdin SV (2010) Population re-sequencing revels local adaptation of Arabidopsis lyrata to serpentine soils. Nature Genetics 42: 260–263. 20101244 10.1038/ng.515

[14] Cheng C, White BJ, Kamdem C, Mockaitis K, Costantini C, et al (2012) Ecological genomics of Anopheles gambiae along a latitudinal cline: a population-resequencing approach. Genetics 190 4 1417–32. 22209907 10.1534/genetics.111.137794 PMC3316653

[15] Futschik A, Schlötterer C (2010) Massively parallel sequencing of pooled DNA samples – the next generation of molecular markers. Genetics 186: 207–218. 20457880 10.1534/genetics.110.114397 PMC2940288

[16] Mackay TF, Richards S, Stone EA, Barbadilla A, Ayroles JF, et al (2012) The *Drosophila melanogaster* Genetic Reference Panel. Nature 482: 173–178. 22318601 10.1038/nature10811 PMC3683990

[17] Huang AM, Rehm EJ, Rubin GM (2009) Quick preparation of genomic DNA from Drosophila. Cold Spring Harb Protoc doi:10.1101/pdb.prot5198. 10.1101/pdb.prot5198 20147141

[18] Li H, Durbin R (2009) Fast and accurate short read alignment with Burrows-Wheeler Transform. Bioinformatics 25: 1754–60. 19451168 10.1093/bioinformatics/btp324 PMC2705234

[19] DePristo M, Banks E, Poplin R, Garimella K, Maguire J, et al (2011) A framework for variation discovery and genotyping using nextgeneration DNA sequencing data. Nature Genetics 43 5 491–498. 21478889 10.1038/ng.806 PMC3083463

[20] McKenna A, Hanna M, Banks E, Sivachenko A, Cibulskis K, et al (2010) The Genome Analysis Toolkit: a MapReduce framework for analyzing next-generation DNA sequencing data. Genome Res 20 9 1297–303 Epub 2010 Jul 19. 20644199 10.1101/gr.107524.110 PMC2928508

[21] Li H, Ruan J, Durbin R (2008) Mapping short DNA sequencing reads and calling variants using mapping quality scores. Genome Res 18 11 1851–8. 18714091 10.1101/gr.078212.108 PMC2577856

[22] R Development Core Team (2011) R: A language and environment for statistical computing. R Foundation for Statistical Computing, Vienna, Austria. ISBN 3-900051-07-0, URL http://www.R-project.org/.

[23] Kofler R, Betancourt AJ, Schlötterer C (2012) Sequencing of Pooled DNA Samples (Pool-Seq) Uncovers Comples Dynamics of Transposable Element Insertions in *Drosophila melanogaster* . PLoS Genet 8 1 e1002487 Doi:10.1371/journal.pgen.1002487. 22291611 10.1371/journal.pgen.1002487 PMC3266889

[24] Lynch M (2009) Estimation of allele frequencies from high-coverage gmenome-sequencing projects. Genetics 182: 295–301. 19293142 10.1534/genetics.109.100479 PMC2674824

[25] Haubold B, Pfaffelhuber P, Lynch M (2010) mlRho a program for estimating the population mutation and recombination rates from shotgun-sequenced diploid genomes. Mol Ecol 19: 277–284. 20331786 10.1111/j.1365-294X.2009.04482.x PMC4870015

[26] Hellmann I, Mang Y, Gu Z, Li P, de la Vega FM, et al (2008) Population genetic analysis of shotgun assemblies of genomic sequences from multiple individuals. Genome Res 18: 1020–1029. 18411405 10.1101/gr.074187.107 PMC2493391

[27] Liu X, Fu Y-X, Maxwell TJ, Boerwinkle E (2010) Estimating population genetic parameters and comparing model goodness-of-fit using DNA sequences with error. Genome Res 20: 101–109. 19952140 10.1101/gr.097543.109 PMC2798822

[28] Clark AG, Whittam TS (1992) Sequencing errors and molecular evolutionary analysis. Mol Biol Evol 9: 744–752. 1630310 10.1093/oxfordjournals.molbev.a040756

[29] Johnson PLF, Slatkin M (2008) Accounting for bias from sequencing error in population genetic estimates. Mol Biol Evol 25: 199–206. 17981928 10.1093/molbev/msm239

[30] Keightley PD, Trivedi U, Thomson M, Oliver F, Kumar S, et al (2009) Analysis of the genome sequences of three Drosophila melanogaster spontaneous mutation accumulation lines. Genome Res 19: 1195–1201. 19439516 10.1101/gr.091231.109 PMC2704435

[31] Ossowski S, Schneeberger K, Lucas-Lledó JI, Warthmann N, Clarke RM, et al (2010) The rate and molecular spectrum of spontaneous mutations in Arabidopsis thaliana. Science 327: 92–94. 20044577 10.1126/science.1180677 PMC3878865

[32] Boitard S, Schlötterer C, Nolte V, Pandey RV, Futschik A (2012) Detecting selective sweeps from pooled next-generation sequencing samples. Mol Biol Evol Doi:10.1093/molbev/mss090. 10.1093/molbev/mss090 PMC3424412 22411855

[33] Ingman M, Gyllensten U (2009) SNP frequency estimation using massively parallel sequencing of pooled DNA. Eur J Hum Genet 17: 383–386. 18854868 10.1038/ejhg.2008.182 PMC2986170

[34] Druley TE, Vallania FLM, Wegner DJ, Varley KE, Knowles OL, et al (2009) Quantification of rare allelic variants from pooled genomic DNA. Nat Methods 6: 263–265. 19252504 10.1038/nmeth.1307 PMC2776647

[35] Wen X, Stephens M (2010) Using linear predictors to impute allele frequencies from summary or pooled genotype data. Ann Appl Stat 4 3 1158–1182. 21479081 10.1214/10-aoas338 PMC3072818

[36] Wang T, Lin C-Y, Rohan TE, Ye K (2010) Re-sequencing of pooled DNA for detecting disease associations with rare variants. Genetic Epid 34: 492–501. 10.1002/gepi.20502 PMC4096227 20578089

[37] Bansal V (2010) A statistical method for the detection of variants from next-generation re-sequencing of DNA pools. ISMB 26: i318–i324. 10.1093/bioinformatics/btq214 PMC2881398 20529923

[38] Kofler R, Orozco-terWengel P, De Maio N, Pandey RV, Nolte V, et al (2011) PoPoolation: A Toolbox for Population Genetic Analysis of Next Generation Sequencing Data from Pooled Individuals. PLoS ONE 6 1 e15925 doi:10.1371/journal.pone.0015925. 21253599 10.1371/journal.pone.0015925 PMC3017084

[39] Wang W, Esbensen Y, Kunke D, Suganthan R, Rachek L, et al (2011) Mitochondrial DNA damage level determines neural stem cell differentiation fate. J Neurosci 31 (26):9746–9751. 21715639 10.1523/JNEUROSCI.0852-11.2011 PMC6623160

