# Supplementary material for: Empirical Validation of Pooled Whole Genome Population Re-Sequencing in Drosophila melanogaster
Source: PLoS One. 2012 Jul 26;7(7):e41901. doi: 10.1371/journal.pone.0041901 (PMC3406057; doi:10.1371/journal.pone.0041901)
Supplement: Supporting Information S1 — Strains used in libraries pooled. (DOC) [file pone.0041901.s001.doc]

**SUPPLEMENTAL FILE S1**

Individual strains used in pooling. (*One strain was later removed from the DGRP database. Its Bloomington ID is shown in place of the RAL_ID.)

Library A: RAL-208, RAL-304, RAL-307, RAL-315, RAL-324, RAL-357, RAL-358, RAL-360, RAL-365, RAL-375, RAL-379, RAL-399, RAL-437, RAL-486, RAL-555, RAL-639, RAL-705, RAL-712, RAL-732, RAL-765, RAL-774, RAL-786

Library B1/2: RAL-40, RAL-318, RAL-897, RAL-398, RAL-392, RAL-338, RAL-802, RAL-721, RAL-642, RAL-535, RAL-879, RAL-272, RAL-426, RAL-237, RAL-195, RAL-367, RAL-136, RAL-491, RAL-790, RAL-370, RAL-310, RAL-42, RAL-399, RAL-324, RAL-774, RAL-375, RAL-712, RAL-360, RAL-365, RAL-786, RAL-765, RAL-358, RAL-555, RAL-335, RAL-380, RAL-820, RAL-852, RAL-391, RAL-427, RAL-517, RAL-461, RAL-371

Library B4: RAL-325, RAL-808, RAL-716, RAL-239, RAL-149, RAL-502, RAL-235, RAL-443, RAL-280, RAL-176, RAL-304, RAL-208, RAL-639, RAL-705, RAL-437, RAL-301, RAL-313, RAL-379, RAL-362, RAL-303, RAL-732, RAL-181, RAL-233, RAL-409, RAL-57, RAL-776, RAL-563, RAL-757, RAL-227, RAL-861, RAL-703, RAL-105, RAL-88, RAL-737, RAL-730, RAL-332, RAL-589, RAL-228, RAL-884, RAL-810, RAL-41, RAL-381, RAL-646, RAL-256, RAL-350, RAL-386, RAL-783, RAL-531, RAL-801, *25123
